# Balance response to levodopa predicts balance improvement after bilateral subthalamic nucleus deep brain stimulation in Parkinson’s disease

**DOI:** 10.1038/s41531-021-00192-9

**Published:** 2021-05-27

**Authors:** Zixiao Yin, Yutong Bai, Liangying Zou, Xin Zhang, Huimin Wang, Dongmei Gao, Guofan Qin, Ruoyu Ma, Kai Zhang, Fangang Meng, Yin Jiang, Anchao Yang, Jianguo Zhang

**Affiliations:** 1 Department of Neurosurgery, Beijing Tiantan Hospital, Capital Medical University, Beijing, China; 2 Beijing Key Laboratory of Neurostimulation, Beijing, China; 3 Department of Functional Neurosurgery, Beijing Neurosurgical Institute, Capital Medical University, Beijing, China

**Keywords:** Neurological manifestations, Parkinson's disease, Outcomes research

## Abstract

The effect of subthalamic nucleus deep brain stimulation (STN-DBS) on balance function in patients with Parkinson’s disease (PD) and the potential outcome predictive factors remains unclear. We retrospectively included 261 PD patients who underwent STN-DBS and finished the 1-month follow-up (M1) assessment in the explorative set for identifying postoperative balance change predictors, and 111 patients who finished both the M1 and 12-month follow-up (M12) assessment in the validation set for verifying the identified factors. Motor and balance improvement were evaluated through the UPDRS-III and the Berg balance scale (BBS) and pull test (PT), respectively. Candidate predictors of balance improvement included age, disease duration, motor subtypes, baseline severity of PD, cognitive status, motor and balance response to levodopa, and stimulation parameters. In the off-medication condition, STN-DBS significantly improved BBS and PT performance in both the M1 and M12, in both datasets. While in the on-medication condition, no significant balance improvement was observed. Higher preoperative BBS response to levodopa was significantly associated with larger postoperative off-medication, but not on-medication, BBS (*p* < 0.001) and PT (*p* < 0.001) improvement in both the M1 and M12. BBS subitems 8, 9, 11, 13, and 14 were the major contributors to the prediction of balance improvement after STN-DBS. STN-DBS improves short-term off-medication, but not on-medication, balance function assessed through BBS and PT. Preoperative BBS response to levodopa best predicts postoperative off-medication balance improvement. For patients who manifested severe balance problems, a levodopa challenge test on BBS or the short version of BBS is recommended.

## Introduction

Axial symptoms severely impact the quality of life in Parkinson’s disease (PD). Falls due to balance problems and postural instability are associated with a higher risk of nursing home or admission to hospitals^1^. Although the subthalamic nucleus deep brain stimulation (STN-DBS) well controls the cardinal symptoms including tremor, rigidity, and bradykinesia in PD, its effect on balance function is still debating. Some researchers indicated that STN-DBS improves postural instability in the short-term, but not in the long-term follow-up^2^. Others found that the probability of fall is increasing despite the improvement in postural instability gait difficulty (PIGD) subscore of unified Parkinson’s disease rating scale (UPDRS) after STN-DBS^3^. One study suggested that STN-DBS may even directly worsen balance capability^4^. In contrast, a recent article published in 2018 showed that STN-DBS did not worsen nor improve balance and postural instability^5^.

Why the influence of STN-DBS on balance is so variable among reports? For one thing, different study designs (prospective vs. retrospective), assessment conditions (off-medication vs. on-medication), and outcome measurements (objective vs. subjective) may lead to different results. For another, the different baseline characteristics of the included patients could cause inconsistent outcomes. It has been suggested that the DBS effect could be associated with factors including age at surgery, motor subtype, baseline severity of symptoms, and response to dopaminergic medication^6,7^. Besides, differences in stimulation settings, e.g., high/low stimulation frequency, could potentially influence the effect of STN-DBS on balance^8^. However, despite these speculations, the associative factor of postoperative balance improvement remains unclear^9,10^. Previous studies generally have a limited sample size, which may hamper the implementation of multifactorial analysis due to the statistical power restrictions. This study, by recruiting a large sample of patients, aims at validating the effect of STN-DBS on balance function in both the off- and on-medication conditions, and further exploring potential predictive factors of postoperative balance improvement after STN-DBS. The answer to this question will help inform the physicians who may or may not gain balance benefit from STN-DBS. The patient with little chance of improving postural stability could be given extra attention and fall-prevention educations right after the operation and during follow-ups.

## Results

### Participants and baseline characteristics

**Fig. 1:**
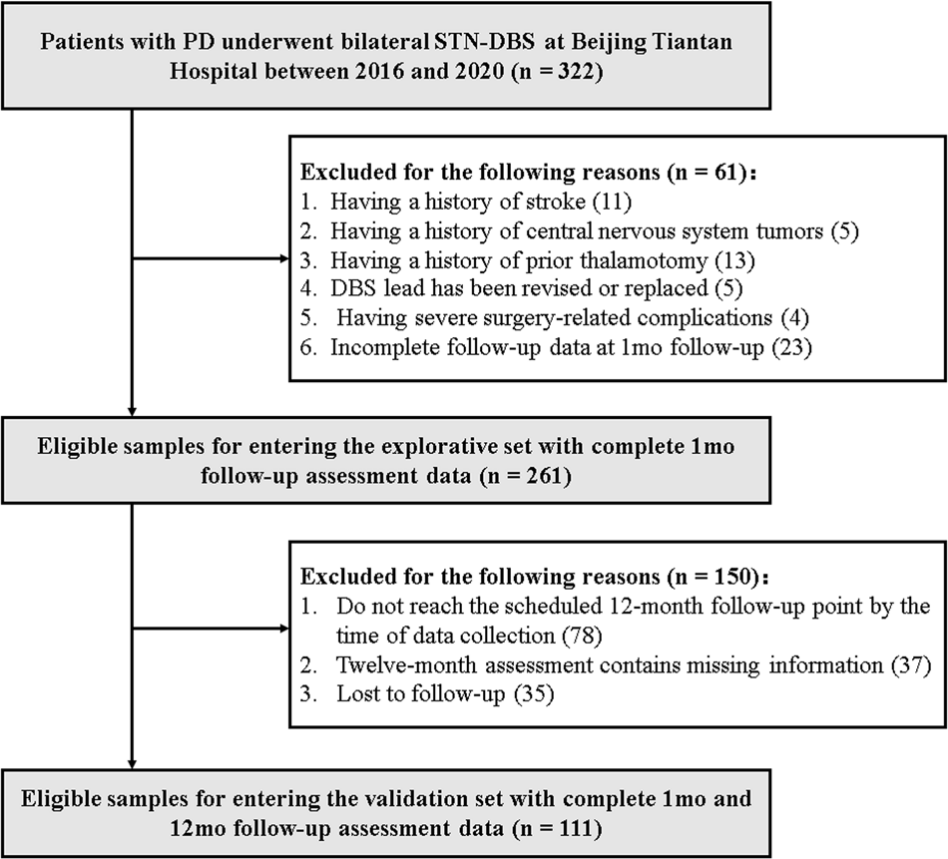
The workflow of participants’ identification in the explorative and the validation datasets. PD, Parkinson’s disease; STN-DBS, subthalamic nucleus deep brain stimulation.

Initially, we identified 322 patients in our dataset who met all the inclusion criteria. After a detailed checking of data and medical records based on exclusion criteria, 61 patients were excluded. Consequently, 261 patients were included in the explorative dataset. Among them, 78 patients did not reach the scheduled 12-month follow-up point by the time of data collection, 37 patients’ assessment data contained missing information, and 35 patients were lost to follow-up, resulting in a total of 111 patients being included in the validation datasets. The detailed workflow of participants’ identification is shown in Fig. 1. For the 261 and 111 patients in the explorative and the validation set, 122 and 65 patients were assessed using UPDRS, and 139 and 46 patients were assessed using Movement Disorder Society-unified Parkinson’s disease rating scale (MDS-UPDRS), respectively. After score conversions, a comparison of baseline characteristics and motor improvement between the 2 datasets are shown in Table 1. There was no significant difference between the 2 sets, except that the patients in the validation set had better on-medication balance performance in Berg balance scale (BBS) at baseline (*p* = 0.016).

**Table 1:** Baseline characteristics and improvement at M1 in the explorative and validation datasets.

	Explorative set (*n* = 261)	Validation set (*n* = 111)	*p* value
Female (%)^a^	41.4	37.8	0.524
Age of onset (yr)	53.3 ± 9.5	54.3 ± 9.4	0.311
Disease duration (yr)	8.9 ± 4.7	9.0 ± 5.6	0.955
Age at surgery (yr)	62.9 ± 8.5	63.9 ± 7.7	0.276
TD/PIGD/MX^a^	96/135/30	37/59/15	0.758
TD/AR/MX^a^	45/167/49	20/63/28	0.325
History of fall^a^	51.2%	49.5%	0.093
History of festinating gait^a^	76.2%	72.1%	0.395
Hoehn-Yahr stage (1/2/3/4/5)^b^	3/51/188/14/5	2/24/75/7/3	0.358
UPDRS-II	21.1 ± 7.7	20.5 ± 7.0	0.549
UPDRS-III ON	26.9 ± 14.4	27.9 ± 14.7	0.548
UPDRS-III OFF	53.7 ± 18.5	56.2 ± 20.7	0.248
UPDRS-III levodopa response (%)	50.7 ± 18.0	50.8 ± 17.3	0.989
BBS ON	49.4 ± 6.4	51.0 ± 4.8	**0.016**
BBS OFF	40.0 ± 12.0	41.3 ± 11.6	0.347
BBS levodopa response (%)	20.8 ± 21.8	18.6 ± 19.7	0.361
PT ON (0/1/2/3/4)^b^	94/118/34/10/5	43/53/13/2/0	0.158
PT OFF (0/1/2/3/4)^b^	17/81/87/58/18	11/31/40/25/4	0.258
LEDD	809.9 ± 588.8	766.6 ± 334.0	0.469
FOG-Q	13.5 ± 8.3	13.0 ± 8.0	0.601
PDQ-39	54.9 ± 22.0	51.6 ± 23.3	0.191
MMSE	26.2 ± 3.7	26.3 ± 3.6	0.783
MoCA	20.8 ± 5.2	20.4 ± 5.1	0.392
UPDRS-III M1 change rate	36.8 ± 27.4%	34.7 ± 27.4%	0.215
BBS M1 change rate	14.8 ± 21.9%	13.3 ± 21.3%	0.346
PT M1 mean difference^b^	0.8 ± 1.0	0.8 ± 1.1	0.445

^a^Chi-square tests.

^b^Mann–Whitney *U* tests.

Unindicated comparisons were conducted using independent *t*-test. Significant comparison is marked in bold.

*M1* 1-month follow-up, *TD* tremor dominant, *PIGD* postural instability and gait disorders, *AR* akinetic-rigid, *MX* mixed type, *ON* on medication, *OFF* off medication, *BBS* Berg balance scale, *PT* pull test, *LEDD* levodopa equivalent daily dose, *FOG-Q* freezing of gait questionnaire, *PDQ-39* Parkinson’s disease questionnaire, *MMSE* mini-mental state examination, *MoCA* Montreal cognitive assessment.

### Motor and balance change at M1 and M12

Comparisons of UPDRS-III, BBS, and pull test (PT) between baseline and follow-ups in the explorative and validation datasets are shown in Table 2. In both the explorative and validation datasets, STN-DBS significantly improved off-medication UPDRS-III, BBS, and PT at M1 and M12 (M12 only in the validation set). However, we observed no significant improvement in the on-medication state in the BBS and PT in follow-ups in both the explorative and validation datasets. No items were significantly changed between M1 and M12.

**Table 2:** Motor and balance change between baseline and follow-ups in the explorative and validation datasets.

	Explorative set (*n* = 261)		Validation set (*n* = 111)				
	M1	Change to M0	M1	Change to M0	M12	Change to M0	M1 vs. M12
UPDRS-III (OFF)	32.1 ± 14.5	**36.8** ± **27.4%**^**a**^	34.2 ± 14.5	**34.7** ± **27.4%**^**a**^	31.7 ± 15.6	**37.1** ± **37.5%**^**a**^	*p* = 0.138
BBS (OFF)	46.2 ± 8.6	**14.8** ± **21.9%**^**a**^	47.1 ± 7.4	**13.3** ± **21.3%**^**a**^	46.8 ± 8.0	**13.1** ± **25.4%**^**a**^	*p* = 0.361
PT (OFF)^b^	1.1 ± 0.8	**0.8** ± **1.0**^**a**^	1.0 ± 0.9	**0.8** ± **1.1**^**a**^	1.1 ± 0.9	**0.7** ± **1.0**^**a**^	*p* = 0.729
UPDRS-III (ON)	19.4 ± 11.2	**25.1** ± **33.7%**^**a**^	21.2 ± 10.1	**21.4** ± **33.7%**^**a**^	19.6 ± 10.1	**25.7** ± **37.6%**^**a**^	*p* = 0.077
BBS (ON)	50.6 ± 7.4	1.3 ± 20.8%	51.5 ± 5.5	2.5 ± 8.7%	50.4 ± 8.8	1.4 ± 11.3%	*p* = 0.108
PT (ON)^b^	0.7 ± 1.0	0.2 ± 0.9	0.6 ± 0.9	0.2 ± 0.8	0.8 ± 0.8	-0.1 ± 1.0	*p* = 0.133

^a^Significant comparison between M1/M12 and M0, *p* < 0.01. Using either paired *t*-test or Wilcoxon signed-rank test.

^b^Change values were presented as mean difference. Comparisons were conducted using Wilcoxon signed-rank test.

Unindicated comparisons were conducted using paired *t*-test. Significant comparisons are marked in bold.

*M0* baseline value, *M1* 1-month follow-up, *M12* 12-month follow-up, *OFF* off medication, *ON* on medication, *BBS* Berg balance scale, *PT* pull test.

### Explorative analysis of balance-improvement predictors

**Fig. 2:**
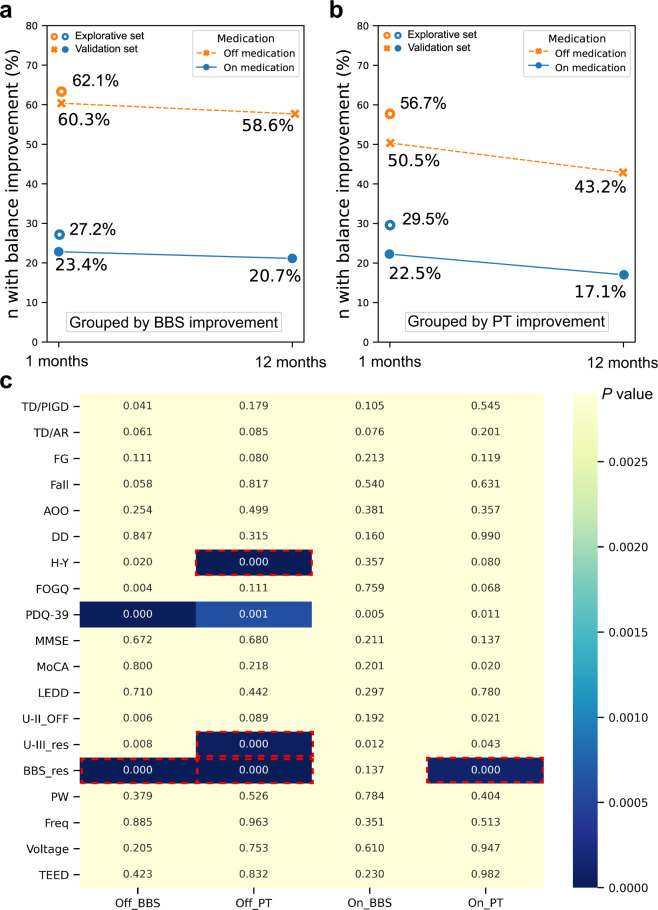
An overview of the percentage of patients being divided into the balance-improvement group according to the BBS and PT criterion. **a** The percentage of patients being divided into the balance-improvement group according to the BBS criteria in both conditions of off- and on-medication in the 1-month follow-up and 12-month follow-up. The orange and the blue circles indicated dichotomies in the explorative set in off- and on-medication conditions, respectively. The orange dotted line and the blue solid line indicated dichotomies in the validation set in off- and on-medication conditions, respectively. **b** The percentage of patients being divided into the balance-improvement group according to the PT criteria in both conditions of off- and on-medication in the 1-month follow-up and 12-month follow-up. **c** Heatmap showing explorative outcomes of balance-improvement predictors. *x*-axis showed 4 different conditions ((medication-on & off) × (criterion-BBS & PT)) based on which balance-improvement dichotomy was made. *y*-axis listed 17 potential predictors. The color of the blocks demonstrated the *p* value in the comparisons of each potential predictor between the balance-improvement group and the non-improvement group, with darker color indicating smaller *p* values. After Bonferroni correction, a *p* < 0.0026 (0.05/19) was considered significant. Blocks with red dotted border were the factors that remained statistically significant in the multivariate regression model. BBS, Berg balance scale; PT, pull test; FG, a history of festinating gait; AOO, age of onset; DD, disease duration; H-Y, Hoehn-Yahr stage; FOG-Q, freezing of gait questionnaire; PDQ-39, Parkinson’s disease questionnaire; MMSE, mini-mental state examination; LEDD, levodopa equivalent daily dose; U-II, UPDRS-II; U-III_res, UPDRS-III response to levodopa; berg_res, Berg balance scale response to levodopa; PW, pulse width; freq, frequency; TEED, total electrical energy delivered; Off_BBS, the dichotomy of off-medication BBS improvement; Off_PT, the dichotomy of off-medication PT improvement; On_BBS, the dichotomy of on-medication BBS improvement; On_PT, the dichotomy of on-medication PT improvement.

Based on the cut-off value in the BBS and PT, the percentage of patients who were dichotomized into the balance-improved group was shown in Fig. 2a, b. Nineteen potential balance-improvement predictors were compared between the balance-improved group and the non-improvement group in different conditions in the explorative set. A heatmap is shown in Fig. 2c where significant predictors were summarized. BBS response to levodopa differed significantly between the balance-improved group and the non-improvement group after correction and remained a significant factor in the multivariate regression (Supplementary Table 3) in predicting balance improvement in 3 out of the 4 conditions. Among other predictors, age, disease duration, motor subtypes, history of fall and festinating gait, baseline cognitive status, baseline freezing of gait questionnaire (FOG-Q), levodopa equivalent daily dose (LEDD), and stimulation parameters did not pass the univariate comparison. Baseline Parkinson’s disease questionnaire (PDQ-39) did not pass the multivariate regression. Baseline Hoehn-Yahr stage and UPDRS-III response to levodopa passed multivariate regression in only 1 out of the 4 conditions. Thus, these factors were not further analyzed in the validation set.

### Preoperative BBS response to levodopa predicts postoperative off-medication balance change

**Fig. 3:**
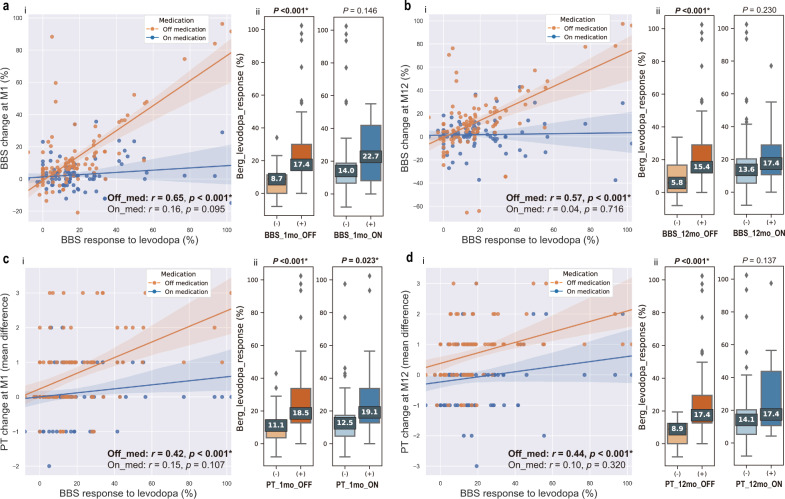
Validation of BBS response to levodopa in predicting balance improvement. **a**-i Scatter plot and least square regression fit curve between BBS response to levodopa and BBS change rate at M1. Orange dots indicate off-medication condition. Blue dots indicate on-medication condition. **a**-ii Box plots showing comparisons of BBS response to levodopa between BBS diagnosed balance-improved groups and non-improvement groups in off-medication conditions (left) and on-medication conditions (right) at M1. Dark orange/blue indicates the balance-improved group and light orange/blue indicates the non-improvement group. **b**-i Scatter plot and least square regression fit curve between BBS response to levodopa and BBS change rate at M12. **b**-ii Box plots showing comparisons of BBS response to levodopa between BBS diagnosed balance-improved groups and non-improvement groups in off-medication conditions (left) and on-medication conditions (right) at M12. **c**-i Scatter plot and least square regression fit curve between BBS response to levodopa and PT change value at M1. **c**-ii Box plots showing comparisons of BBS response to levodopa between PT diagnosed balance-improved groups and non-improvement groups in off-medication conditions (left) and on-medication conditions (right) at M1. **d**-i Scatter plot and least square regression fit curve between BBS response to levodopa and PT change value at M12. **d**-ii Box plots showing comparisons of BBS response to levodopa between PT diagnosed balance-improved groups and non-improvement groups in off-medication conditions (left) and on-medication conditions (right) at M12. Significant *p* values are highlighted in bold. For boxplots, minimum and maximum are represented by lower and upper whiskers, respectively. The box signifies the first and the third quartile, and the median is represented by the marked center value within the box. BBS, Berg balance scale; PT, pull test; M1, 1-month follow-up; M12, 12-month follow-up.

The relation between BBS response to levodopa and balance change (BBS and PT) was validated in 2 conditions (on-med and off-med), at 2 timepoints (M1 and M12), and using 2 approaches (continuous linear correlation and binary subgroup analysis) in the validation set. We found that preoperative BBS response to levodopa was positively correlated with postoperative balance change in off-medication conditions, but not in on-medication conditions (Fig. 3 ai–di); preoperative BBS response to levodopa was significantly higher in the balance-improved groups than in the non-improvement groups in off-medication conditions (Fig. 3 aii–dii), but not in on-medication conditions with only 1 exception (Fig. 3 cii), where the difference between groups was also significant in the on-medication condition.

Given that the scoring systems of PT in UPDRS and MDS-UPDRS are not entirely consistent (e.g., the patient scoring 1 point in UPDRS could score 1 or 2 points in MDS-UPDRS)^11^, PT results were also validated in patients assessed using UPDRS (*n* = 65) and using MDS-UPDRS (*n* = 46), separately. The results in both subgroups were identical to what is observed when the patients were combined analyzed (Supplementary Fig. 1). Specifically, for the 46 patients assessed using MDS-UPDRS, significant correlation was observed between baseline BBS response to levodopa and off-medication PT change at M1 (*r* = 0.44, *p* = 0.002) and M12 (*r* = 0.36, *p* = 0.015). For the 65 patients assessed using UPDRS, significant correlation was observed between baseline BBS response to levodopa and off-medication (*r* = 0.41, *p* < 0.001) and on-medication (*r* = 0.26, *p* = 0.027) PT change at M1, and off-medication PT change at M12 (*r* = 0.52, *p* < 0.001).

### Relative importance of BBS subitems

**Fig. 4:**
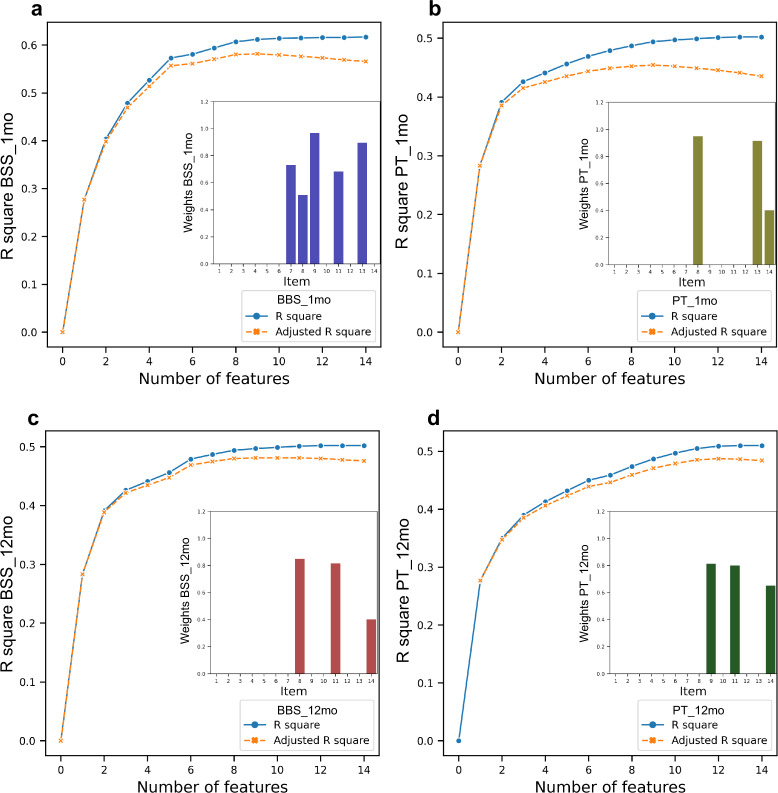
Model selection by backward feature elimination in exploring the relative importance of subitems of BBS in off-medication conditions. Blue curves represented R-square. Orange curves represented the adjusted R-square. The relative weights of the significant features are shown on the bottom right of each plot. **a** Regression of data based on the dichotomy of BBS improvement in the M1. **b** Regression of data based on the dichotomy of BBS improvement in the M12. **c** Regression of data based on the dichotomy of PT improvement in the M1. **d** Regression of data based on the dichotomy of PT improvement in the M12. The subitems of BBS: item-1, sitting to standing; item-2, standing unsupported; item-3, sitting unsupported; item-4, standing to sitting; item-5, transfers; item-6, standing with eyes closed; item-7, standing with feet together; item-8, reaching forward with outstretched arm; item-9, retrieving object from floor; item-10, turning to look behind; item-11, turning 360°; item-12, placing alternate foot on stool; item-13, standing with one foot in front; and item-14, standing on one foot. BBS, Berg balance scale; PT, pull test; M1, 1-month follow-up; M12, 12-month follow-up.

Further, we explored the relative importance of BBS subitems in predicting balance improvement in the off-medication condition. Figure 4 showed that in M1, BBS items 7, 8, 9, 11, 13 significantly contributed to the prediction of BBS improvement, and BBS items 8, 13, 14 contributed to the prediction of PT improvement. In M12, BBS items 8, 11, 14 significantly contributed to the prediction of BBS improvement, and BBS items 9, 11, 14 contributed to the prediction of PT improvement. Overall, items 8 (reaching forward with outstretched arm), 9 (retrieving object from floor), 11 (turning 360°), 13 (standing with one foot in front), and 14 (standing on one foot) had been significant contributors at least two times. We considered these items as high-contributor items.

## Discussion

This study explored the effect of STN-DBS on balance function in both the on- and off-medication conditions. We found that in the off-medication state, STN-DBS significantly improved BBS and PT score in both the M1 and M12, while in the on-medication state, STN-DBS may not provide extra benefits than medicine in improving balance at either timepoint. Possible outcome predictors were also studied. Motor subtypes and stimulation parameters, which were expected to be influential to balance outcome^8,12^, were not found to be significantly associated with the BBS or PT improvement after STN-DBS. Instead, preoperative response to levodopa in the BBS was significantly different between the balance-improved and non-improvement groups. Regression analysis showed that several subitems of BBS were the major contributors to the correlation.

Identical to our results, several previous studies also found that STN-DBS can improve off-medication balance function employing different measurements^13^. However, on-medication outcomes were debating. Li et al. observed a significant balance improvement in both the on- and off-medication conditions^14^. While Brandmeir et al. did not observe the improvement^5^. Zaidel et al. indicated that compared with tremor and rigidity, the axial and balance problems were least responsive to DBS in the on-medication assessment^15^. We found that STN-DBS did not improve on-medication balance function assessed by either BBS or PT in M1 and M12, despite that on-medication UPDRS-III was significantly improved. Some authors attributed the lesser benefit in balance to the stimulation parameter, as the stimulation parameters may be programmed to alleviate cardinal symptoms, rather than the balance symptoms^2^. Other authors proposed that postural instability may respond naturally different to STN-DBS compared to tremor and rigidity, evident by the fact that tremor and rigidity are improved almost instantly after turning on the stimulation, while balance change could delay for a few hours^16^.

Compared to the typical Parkinsonism symptoms such as tremor and rigidity, postural instability normally had a worse response to levodopa ^10^. Occasionally, after taking medication, balance function could even get worse^17^. In our study, the preoperative BBS response to levodopa was only around 20%, far lower than the over 50% improvement in the UPDRS-III. However, despite the relatively low response, this value could potentially help predict the short-term balance benefit brought by STN-DBS. We found that preoperative BBS response to levodopa was significantly different between the balance-improved and non-improvement groups. This suggested that STN-DBS may better improve symptoms that are sensitive to medication, and less likely to improve the insensitive ones^9^. We assumed the following reasons could explain this. First, postural control of the body calls for the coordination of limb and trunk muscles. Thus, the improvement of cardinal signs of PD could contribute to the improvement of balance function. For example, when the velocity and quality of the stepping are improved, the ability to resist external interference will also improve during walking^18^. In addition, balance function will also improve when the mobility of protective arms is increased, which serves to maintain postural stability following sudden external balance perturbations^19^. These symptoms seem to respond to both STN-DBS and medication. Second, STN-DBS and levodopa may have some overlapping effects on several subdomains of balance adjustment^20^. Anticipatory postural adjustments, the ability to activate the postural muscles before movements in anticipation of the destabilizing forces, may respond similarly to levodopa and DBS^13^. Besides, as reported by George et al., automatic postural responses were improved both after taking medication before surgery and turning on stimulation after surgery^21^. Third, some other symptoms that may indirectly impact balance, such as posture abnormality^22^ and emotional problems^23^, could also be alleviated by both the DBS and medication. Fasano et al.^10^. divided the preoperative balance problems into levodopa-sensitive type, levodopa-insensitive type, and levodopa-induced type. DBS may provide the best benefit to the levodopa-sensitive balance problems, and less likely to improve levodopa-insensitive symptoms. But it should be noted that the response of balance function to DBS and levodopa is not completely the same. Rocchi et al. found that stimulation and levodopa differ in their influence on postural sway in stance^24^. Shivitz et al. also found that in the sensory aspect of postural control, DBS and medication may play different roles^25^.

It is suggested that the balance function can be divided into multiple subdomains. May et al. reported that stimulation and medication improved 4 out of the 6 balance subsections in PD, including biomechanical constraints, stability limits, anticipatory postural adjustments, and sensory orientation^13^. In our study, balance capability was mainly assessed through BBS, which focused more on the anticipatory postural adjustments. We found that several subitems of the BBS already contributed to most of the predictive ability in regression models. The high-contributor items are item 8 (reaching forward with outstretched arms), item 9 (retrieving object from floor), item 11 (turning 360°), item 13 (standing with one foot in front), and item 14 (standing on one foot). We recommended that comparing the performance of these items before and after taking medication in the preoperative assessment is meaningful. Although BBS is a well-established scale in balance function evaluation, its complexity both in implementation and scoring may hamper its widespread utility. Many efforts have been made to shorten the BBS. Chou et al. indicated that the BBS could be shortened to a form containing 7 three-level items, without losing its psychometric properties^26^. Interestingly, 4 out of the 5 high-contributor items found in our study were included in the 7-item shortened BBS. Considering the potential to predict balance improvement after STN-DBS, a levodopa challenge test on BBS or the short version of the BBS should be conducted in patients who exhibited severe postural control problems before the surgery.

Our study has several limitations that should be noted. First, for study design, we set no control groups (medication only or sham stimulation) in this study. Thus, the potential influence of the placebo effect of STN-DBS on balance function cannot be ruled out in the analysis of balance improvement. A best medication treatment group or a sham stimulation group would strongly enhance the evidence level of this study. Second, the motor function of included patients was assessed using different scales, i.e., UPDRS and MDS-UPDRS, which may introduce confounding factors. We employed validated formulas^27^ to convert the total score between the 2 scales and also validated the subitem results separately in UPDRS and MDS-UPDRS groups. Given that most of our analyses were the comparisons between different conditions within subjects, and for a single patient the applied scale is always consistent, the influence of employing different evaluation scales could be alleviated. Third, factor screening was conducted based on data collected at M1, during which DBS effects may not have reached a steady state. But the comparisons between M1 and M12 showing no difference, indicating the results at M1 is valid. Besides, data at M1 may better reflect the acute effect of STN-DBS as the influence of disease progression is eliminated. Fourth, the identified predictor is validated only in the 1-year follow-up. Given that the PIGD score may worsen significantly 2 years after surgery^2^, future studies analyzing long-term balance-improvement predictors could be meaningful. At last, the volumes of tissue activated in the STN were not analyzed in our study, because high-resolution preoperative MRI and matched thin-layer postoperative CT necessary for accurate reconstruction of the contact within the STN were lacking in a proportion of patients, especially the ones enrolled in early years. Future studies reporting this information together with stimulation settings would contribute to a better understanding of how stimulation of the STN affects balance function.

In patients with PD, bilateral STN-DBS significantly improves balance function evaluated through BBS and PT in the off-medication assessment, but not in the on-medication assessment in M1 and M12. Preoperative BBS response to levodopa best predicts postoperative off-medication balance improvement. BBS subitems 8, 9, 11, 13, and 14 are the high-contributor items in the prediction. A levodopa challenge test on BBS or the short version of BBS should be conducted before surgery for patients with postural control problems.

## Methods

### Participant identification

We retrospectively collected data from patients who were diagnosed as PD and underwent bilateral STN-DBS at Beijing Tiantan Hospital between 2016 and 2020. Inclusion criteria included: (1) PD was diagnosed based on the UK brain bank criteria; (2) bilateral STN-DBS surgery was performed, and (3) patients underwent complete clinical assessment at 1 month or 12 months after surgery. Excluded criteria included: (1) having a history of other diseases that could influence balance such as stroke and central nervous system tumors (confirmed by MRI and/or CT scan); (2) having a history of prior thalamotomy; (3) DBS lead has been revised or replaced; (4) having severe surgery-related complications such as cerebral hemorrhage and hemiplegia; and (5) assessment data were incomplete. This study was conducted under the approval of the IRB of Beijing Tiantan Hospital (KY 2020-139-01). All patients provided written informed consent.

### Standard surgical and clinical assessment procedures

The standard surgical procedure was conducted as previously reported^28^. Briefly, DBS electrodes (model 3389, Medtronic, USA, or model L301, Pins Medical, China) were implanted using a Leksell micro-stereotactic system (Elekta Instrument AB, Stockholm, Sweden) under local anesthesia. Intraoperative microelectrode recording and macro-stimulation tests were performed for trajectory selection. Then, an implantable pulse generator (IPG) connected to the electrodes was implanted in the subclavicular area under general anesthesia. Electrode position was verified through CT scanning or lead reconstruction. Detailed clinical assessment was conducted including motor function evaluation (e.g., UPDRS or MDS-UPDRS), symptom-related scales (e.g., BBS, FOG-Q, and PDQ-39), and cognitive test batteries ((e.g., the mini-mental state examination (MMSE), and the Montreal cognitive assessment (MoCA)). Clinical assessment was conducted by 2 movement disorders specialists (L.Z. and H.W.) in 2 preoperative conditions (off-medication and on-medication), and 2 postoperative conditions (off-medication/on-stimulation and on-medication/on-stimulation) at 1-month follow-up (M1) or 12-month follow-up (M12). At M1 and M12, patients return to the hospital for switching on pulse generator and/or adjusting stimulation parameters at the outpatient programming service. After a 3–7 days observation period (depending on the patients’ schedule), patients were then introduced to the evaluation center for an overall motor and neuropsychological assessment in both the off- and on-medication states. We performed off-medication assessments after at least a 12 h withdrawal of dopaminergic medications, and on-medication assessments approximately 1 h after taking medication. All motor evaluations were videotaped after obtaining the consent of the patients. We recorded LEDD and stimulation settings at every assessment time point.

### Balance measurement and improvement dichotomy

Balance capability was evaluated utilizing the BBS and PT (UPDRS item-30 or MDS-UPDRS item-3.12). BBS is a 14-item scale assessing balance function in older adults or individuals with conditions prone to fall such as PD^29^. Each item of BBS contains 5 grades with a range of 0–4; “4” indicates the highest level of balance function and “0” indicates the lowest^30^. The total score is 56. Supplementary Table 1 shows the 14 subitems of BBS. PT is a quick examination contained in the standard motor assessment of PD in testing postural instability. PT is done by pulling on both shoulders of the patient and observing the patient’s reaction^11^. Under the scoring system of the UPDRS, if the patient takes less than 2 steps to maintain balance, he scores 0. If the patient takes more than 2 steps but stops himself from falling, he scores 1. If the patient falls after pulling, he scores 2. If the patient tends to fall spontaneously even without pulling, he scores 3. If the patient cannot stand without assistance, he scores 4. Based on the BBS and PT improvement, patients were dichotomized into the balance-improved group and the non-improvement group. For BBS, a minimal detectable change (MDC) was employed to determine the cut-off^31^. An improvement of 4 points in the follow-up was regarded as a meaningful improvement if the patients scored within 45–56 at baseline assessment, 5 points if scored within 35–44, 7 points if scored within 25–34, and 5 points if scored within 0–24 on the BBS^31^. Notably, this criterion was established for elderly patients (i.e., over 65 years), who can be slightly older than our population. To the best of our knowledge, an MDC for PT has not been established^32^. Based on that, the minimal clinically important change in UPDRS/MDS-UPDRS motor score is around 3 points^33,34^, and that authors have employed 1 point as a cut-off value for determining minimal detectable improvement for UPDRS/MDS-UPDRS subitems^35,36^, we regarded an improvement of ≥1 point in PT as a meaningful improvement.

### Potential balance-improvement predictors

We collected information on potential predictors of balance improvement, including the age of onset, disease duration, motor subtypes, history of fall, history of festinating gait, baseline score of FOG-Q, PDQ-39, MMSE, MoCA, UPDRS-II, and Hoehn-Yahr stage, UPDRS-III and BBS response to levodopa, and stimulation parameters in the follow-ups including stimulation pulse width, frequency, voltage, and the total electrical energy delivered (TEED) [calculated as TEED = (voltage^2^ × pulse width × frequency)/impedance)]^37^. History of fall was defined as at least 1 time of “unintentionally falling not due to external force or a loss of consciousness since the onset of PD^38^.” A history of festinating gait was defined as the symptom of “the propensity to lean forward becoming invincible during walking^39^” is exhibiting or has exhibited. The Hoehn-Yahr stage was collected in the OFF condition only. UPDRS-III response to levodopa was calculated as ((Off-score – On-score)/Off-score × 100%). BBS response to levodopa was calculated as ((On-score – Off-score)/Off-score × 100%). Notably, since some patients were assessed using UPDRS while others were assessed using MDS-UPDRS (with the scale employed for every single patient being consistent across follow-ups), UPDRS-II and -III were uniformly converted to MDS-UPDRS-II and -III employing previously reported methods^27^, which has been proved to be valid. We converted only UPDRS-II and UPDRS-III because the method is valid only for these two parts. The conversion formulas are shown in Supplementary Table 2.

We employed two commonly used methods (tremor dominant-postural instability and gait disorders (TD/PIGD) and tremor dominant-akinetic-rigid (TD/AR)) to conduct motor subtype classifications^40,41^. Given that the UPDRS and MDS-UPDRS were both used in our clinical assessment, classifications based on both scales were introduced. For the TD/PIGD classification based on UPDRS, the ratio of the mean tremor score (UPDRS 2.16, 3.20, and 3.21) to the mean PIGD score (UPDRS 2.13–2.15 and 3.29–3.30) was calculated. A ratio of ≥1.5 indicated TD subtype and ≤1.0 indicated PIGD subtype^40^. For the TD/PIGD classification based on MDS-UPDRS, we also calculate the ratio of the mean tremor score (MDS-UPDRS 2.10, and 3.15–3.18) to the mean PIGD score (MDS-UPDRS 2.12–2.13, and 3.10–3.12). A ratio of ≥1.15 indicated TD subtype and ≤0.90 indicated PIGD subtype^42^. For the TD/AR classification, since we did not find a validated calibration that can convert the cut-off ratio in UPDRS to that in MDS-UPDRS, we chose to use the same items in MDS-UPDRS to substitute the corresponding items in UPDRS. The mean tremor score was calculated as an average of UPDRS 3.20–3.21, or MDS-UPDRS 3.15, 3.17. Mean AR score was calculated as an average of UPDRS 3.22–3.27, 3.31 or MDS-UPDRS 3.3–3.6, 3.8–3.9, 3.14. A ratio of >1.0 indicated TD subtype and <0.80 indicated AR subtype^41^.

### Explorative and validation datasets

Two datasets were included in this study. The explorative dataset serves to exploratively analyze the potential balance-improvement predictors after acute DBS. This dataset has a large sample size but contains data in M1 only. The validation dataset was smaller in sample size, but had complete data in both the M1 and M12, serving to validate the factor identified in the explorative dataset in longer follow-ups. This strategy explored the balance response after both the acute and chronic STN stimulation. M1 was chosen because disease progression would not play a role at this timepoint, and stimulation parameters are more variable at M1, helping study the influence of stimulation settings on balance. Besides, factor analysis usually requires a large sample size (e.g., 3–20 times the number of the variables^43^). The sample size in M1 is large enough to provide sufficient statistical power.

### Statistical analysis

Data were presented as mean ± SD when under normal distribution and median (range) when under skewed distribution. Ordinal data were presented as the count number in each rank. Inter-group comparisons of demographic data between patients in the 2 sets were conducted using independent *t*-tests for continuous variables, Mann–Whitney *U* test for ordinal variables and Chi-square test for binary variables. Intra-group comparisons of motor and balance capability between baseline and follow-ups were conducted using paired *t*-test or Wilcoxon signed-rank test. We employed both the uni- and multivariate analysis to explore potential balance-improvement predictors in the explorative set. In the univariate exploration, patients were classified into the balance-improved group and the non-improvement group in 4 conditions ((medication-on & off) × (criterion-BBS & PT)). In each condition, we compared candidate predictors between groups with *p* values corrected for multiple comparisons. Significant factors identified in the univariate analysis were eligible for entering the multivariate logistic model. Then, we validated the most significant factors identified in the explorative set in a validation set using methods of correlation analysis and subgroup analysis in both timepoints, M1 and M12. Further, the relative importance of BBS subitems was also explored. The 14 BBS subitems were put into a multiple regression model in predicting balance improvement adopting a backward elimination strategy^44^. A standardized logistic regression coefficient was calculated for each item to measure relative weight. Model performance was assessed through R-square and adjusted R-square, which adjusted for the number of features entering the model to avoid overfitting^45^. A two-tailed *p* value smaller than 0.05 was considered significant. All statistical analysis was conducted using SPSS 24 (IBM, Chicago, IL, USA) and Python 3.

### Reporting summary

Further information on research design is available in the Nature Research Reporting Summary linked to this article.

## Supplementary information

Supplementary information.

Reporting summary.

## Data Availability

The data that support the findings of this study are available from the corresponding author upon reasonable request after consideration and approval of local IRB.

## Code availability

The code used to conduct statistics in this study is available from the corresponding author upon reasonable request.
