# Supplementary material for: Balance response to levodopa predicts balance improvement after bilateral subthalamic nucleus deep brain stimulation in Parkinson’s disease
Source: NPJ Parkinsons Dis. 2021 May 27;7:47. doi: 10.1038/s41531-021-00192-9 (PMC8160136; doi:10.1038/s41531-021-00192-9)
Supplement: Supplementary file 1 — Supplementary information. [file 41531_2021_192_MOESM1_ESM.pdf]

SUPPLEMENTARY INFORMATION

SUPPLEMENTAL TABLES

Supplementary Table 1. Subitems in the Berg Balance Scale

Item number	Item name
Item 1	Sitting to standing
Item 2	Standing unsupported
Item 3	Sitting unsupported
Item 4	Standing to sitting
Item 5	Transfers
Item 6	Standing with eyes closed
Item 7	Standing with feet together
Item 8	Reaching forward with outstretched arm
Item 9	Retrieving object from floor
Item 10	Turning to look behind
Item 11	Turning 360 degrees
Item 12	Placing alternate foot on stool
Item 13	Standing with one foot in front
Item 14	Standing on one foot

Supplementary Table 2. Formulas for converting UPDRS-II and -III score to MDS-UPDRS-II and -III score in different Hoehn-Yahr groups.

Hoehn-Yahr stage	MDS-UPDRS II	MDS-UPDRS III
I/II	(UPDRS II × 1.1) + 0.2	(UPDRS III × 1.2) + 2.3
III	(UPDRS II × 1.0) + 1.5	(UPDRS III × 1.2) + 1.0
IV/V	(UPDRS II × 1.0) + 4.7	(UPDRS III × 1.1) + 7.5

UPDRS: Unified PD Rating Scale; MDS-UPDRS: Movement Disorder Society Unified PD Rating Scale.

Supplementary Table 3. Multivariate logistic regression in predicting balance improvement in the explorative set.

	B	SE	OR	95% CI	p value
M1 Off-medication BBS					
PDQ-39	0.010	0.008	1.010	0.995-1.025	0.201
BBS response to levodopa	0.134	0.021	1.144	1.098-1.191	**<0.001**
M1 Off-medication PT					
PDQ-39	0.005	0.007	1.005	0.991-1.019	0.510
Hoehn-Yahr stage	1.126	0.505	3.085	1.147-8.293	**0.026**
UPDRS-III response to levodopa	0.028	0.009	1.028	1.011-1.046	**0.001**
BBS response to levodopa	0.062	0.013	1.064	1.037-1.092	**<0.001**

Note: above results are obtained through the Enter regression method. We also employed the Backward

and Forward stepwise methods, which did not change the results.

M1: 1-month follow-up; BBS: Berg balance scale; PT: pull test; PDQ-39: Parkinson's disease questionnaire.

SUPPLEMENTAL FIGURES

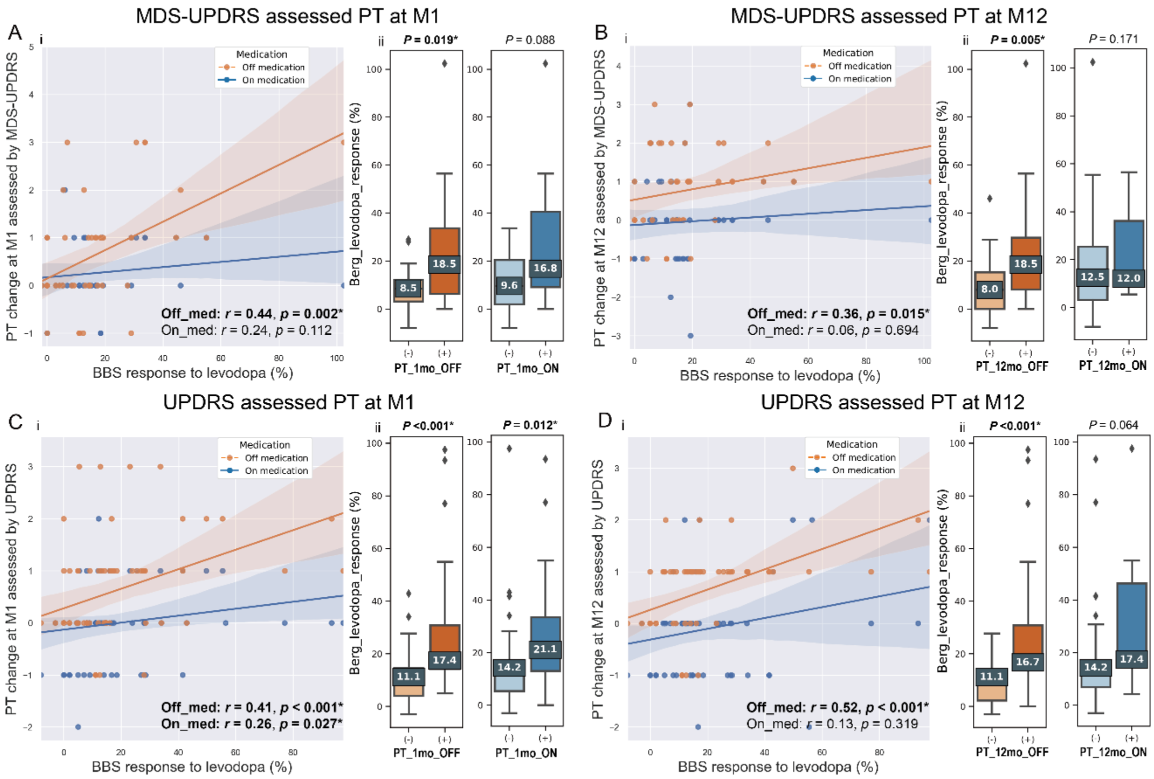

Supplementary Figure 1. Separate validations of BBS response to levodopa in predicting PT improvement in patients assessed using UPDRS and MDS-UPDRS. A-i Scatter plot and least square regression fit curve between BBS response to levodopa and PT change value at M1 in patients assessed using MDS-UPDRS. Orange dots indicate off-medication condition. Blue dots indicate on-medication condition. A-ii Box plots showing comparisons of BBS response to levodopa between PT diagnosed balance-improved groups and non-improvement groups in off-medication conditions (left) and on-medication conditions (right) at M1. Dark orange/blue indicates the balance-improved group and light orange/blue indicates the non-improvement group. B-i Scatter plot and least square regression fit curve between BBS response to levodopa and PT change value at M12 in patients assessed using MDS-UPDRS. B-ii Box plots showing comparisons of BBS response to levodopa between PT diagnosed

balance-improved groups and non-improvement groups in off-medication conditions (left) and on-medication conditions (right) at M12. C-i Scatter plot and least square regression fit curve between BBS response to levodopa and PT change value at M1 in patients assessed using UPDRS. C-ii Box plots showing comparisons of BBS response to levodopa between PT diagnosed balance-improved groups and non-improvement groups in off-medication conditions (left) and on-medication conditions (right) at M1. D-i Scatter plot and least square regression fit curve between BBS response to levodopa and PT change value at M12 in patients assessed using UPDRS. D-ii Box plots showing comparisons of BBS response to levodopa between PT diagnosed balance-improved groups and non-improvement groups in off-medication conditions (left) and on-medication conditions (right) at M12. Significant p values are highlighted in bold. For boxplots, minimum and maximum are represented by lower and upper whiskers, respectively. The box signifies the first and the third quartile, and the median is represented by the marked centre value within the box.

BBS: Berg balance scale; PT: pull test; M1: 1-month follow-up; M12: 12-month follow-up.
