# Supplementary material for: Balance response to levodopa predicts balance improvement after bilateral subthalamic nucleus deep brain stimulation in Parkinson’s disease
Source: NPJ Parkinsons Dis. 2021 May 27;7:47. doi: 10.1038/s41531-021-00192-9 (PMC8160136; doi:10.1038/s41531-021-00192-9)
Supplement: Supplementary file 2 — Reporting summary. [file 41531_2021_192_MOESM2_ESM.pdf]

nature research

Corresponding author(s): DBPR NPJPARKD-00629R1
Last updated by author(s): Apr 23, 2021

Reporting Summary

Nature Research wishes to improve the reproducibility of the work that we publish. This form provides structure for consistency and transparency in reporting. For further information on Nature Research policies, see our Editorial Policies and the Editorial Policy Checklist.

Statistics

For all statistical analyses, confirm that the following items are present in the figure legend, table legend, main text, or Methods section.

n/a	Confirmed	
☐	☒	The exact sample size (*n*) for each experimental group/condition, given as a discrete number and unit of measurement
☐	☒	A statement on whether measurements were taken from distinct samples or whether the same sample was measured repeatedly
☐	☒	The statistical test(s) used AND whether they are one- or two-sided<br>*Only common tests should be described solely by name; describe more complex techniques in the Methods section.*
☐	☒	A description of all covariates tested
☐	☒	A description of any assumptions or corrections, such as tests of normality and adjustment for multiple comparisons
☐	☒	A full description of the statistical parameters including central tendency (e.g. means) or other basic estimates (e.g. regression coefficient) AND variation (e.g. standard deviation) or associated estimates of uncertainty (e.g. confidence intervals)
☐	☒	For null hypothesis testing, the test statistic (e.g. *F*, *t*, *r*) with confidence intervals, effect sizes, degrees of freedom and *P* value noted<br>*Give P values as exact values whenever suitable.*
☒	☐	For Bayesian analysis, information on the choice of priors and Markov chain Monte Carlo settings
☒	☐	For hierarchical and complex designs, identification of the appropriate level for tests and full reporting of outcomes
☐	☒	Estimates of effect sizes (e.g. Cohen's *d*, Pearson's *r*), indicating how they were calculated

Our web collection on statistics for biologists contains articles on many of the points above.

Software and code

Policy information about availability of computer code

Data collection	Data were collected using Microsoft Excel.
Data analysis	All statistical analysis was conducted using SPSS 24 (IBM, Chicago, IL, USA) and Python 3.

For manuscripts utilizing custom algorithms or software that are central to the research but not yet described in published literature, software must be made available to editors and reviewers. We strongly encourage code deposition in a community repository (e.g. GitHub). See the Nature Research guidelines for submitting code & software for further information.

Data

Policy information about availability of data

All manuscripts must include a data availability statement. This statement should provide the following information, where applicable:
- Accession codes, unique identifiers, or web links for publicly available datasets
- A list of figures that have associated raw data
- A description of any restrictions on data availability

The data that support the findings of this study are available from the corresponding author upon reasonable request after consideration and approval of local IRB.

Field-specific reporting

Please select the one below that is the best fit for your research. If you are not sure, read the appropriate sections before making your selection.

☒ Life sciences ☐ Behavioural & social sciences ☐ Ecological, evolutionary & environmental sciences

For a reference copy of the document with all sections, see nature.com/documents/nr-reporting-summary-flat.pdf

Life sciences study design

All studies must disclose on these points even when the disclosure is negative.

Sample size	We included 261 patients in this study. Since this is a retrospective study, no predetermination of sample size was conducted. We collected all patients received DBS surgery in our center between 2016-2020.
Data exclusions	The exclusion criteria were pre-established.<br>Excluded criteria included: (1) having a history of other diseases that could influence balance such as stroke and central nervous system tumors (confirmed by MRI and/or CT scan); (2) having a history of prior thalamotomy; (3) DBS lead has been revised or replaced; (4) having severe surgery-related complications such as cerebral hemorrhage and hemiplegia and (5) assessment data were incomplete.
Replication	All the replications are successful.
Randomization	We cannot perform randomization because this is a retrospective study. The covariate was controlled by multi-variate regreesion.
Blinding	We cannot perform blinding because this is a retrospective study.

Reporting for specific materials, systems and methods

We require information from authors about some types of materials, experimental systems and methods used in many studies. Here, indicate whether each material, system or method listed is relevant to your study. If you are not sure if a list item applies to your research, read the appropriate section before selecting a response.

Materials & experimental systems

n/a	Involved in the study	
☒	☐	Antibodies
☒	☐	Eukaryotic cell lines
☒	☐	Palaeontology and archaeology
☒	☐	Animals and other organisms
☐	☒	Human research participants
☒	☐	Clinical data
☒	☐	Dual use research of concern

Methods

n/a	Involved in the study	
☒	☐	ChIP-seq
☒	☐	Flow cytometry
☒	☐	MRI-based neuroimaging

Human research participants

Policy information about studies involving human research participants

Population characteristics	We included 261 patients diagnosed with Parkinson's Disease. The mean age of the population is 62.9. Female percent is 41.4%. All of them received bilateral subthalamic nucleus deep brain stimulation surgery.
Recruitment	We retrospectively collected data from patients who were diagnosed as PD and underwent bilateral STN-DBS at Beijing Tiantan Hospital between 2016 and 2020. Inclusion criteria included: (1) PD was diagnosed based on the UK brain bank criteria; (2) bilateral STN-DBS surgery was performed, and (3) patients underwent complete clinical assessment at 1 month or 12 months after surgery.
Ethics oversight	This study was conducted under the approval of the IRB of Beijing Tiantan Hospital (KY 2020-139-01).

Note that full information on the approval of the study protocol must also be provided in the manuscript.
